# The Incredible Journey of Begomoviruses in Their Whitefly Vector

**DOI:** 10.3390/v9100273

**Published:** 2017-09-24

**Authors:** Henryk Czosnek, Aliza Hariton-Shalev, Iris Sobol, Rena Gorovits, Murad Ghanim

**Affiliations:** 1 Institute of Plant Sciences and Genetics in Agriculture, Robert H. Smith Faculty of Agriculture, Food and Environment, The Hebrew University of Jerusalem, Rehovot 7610001, Israel; aliza.hariton@mail.huji.ac.il (A.H.-S.); iris.sobol@mail.huji.ac.il (I.S.); rena.gorovits@mail.huji.ac.il (R.G.); 2 Department of Entomology, Agricultural Research Organization, Volcani Center, HaMaccabim Road 68, Rishon LeZion 7505101, Israel; ghanim@volcani.agri.gov.il

**Keywords:** begomovirus, TYLCV, whitefly, transcription and replication, autophagy

## Abstract

Begomoviruses are vectored in a circulative persistent manner by the whitefly *Bemisia tabaci.* The insect ingests viral particles with its stylets. Virions pass along the food canal and reach the esophagus and the midgut. They cross the filter chamber and the midgut into the haemolymph, translocate into the primary salivary glands and are egested with the saliva into the plant phloem. Begomoviruses have to cross several barriers and checkpoints successfully, while interacting with would-be receptors and other whitefly proteins. The bulk of the virus remains associated with the midgut and the filter chamber. In these tissues, viral genomes, mainly from the tomato yellow leaf curl virus (TYLCV) family, may be transcribed and may replicate. However, at the same time, virus amounts peak, and the insect autophagic response is activated, which in turn inhibits replication and induces the destruction of the virus. Some begomoviruses invade tissues outside the circulative pathway, such as ovaries and fat cells. Autophagy limits the amounts of virus associated with these organs. In this review, we discuss the different sites begomoviruses need to cross to complete a successful circular infection, the role of the coat protein in this process and the sites that balance between virus accumulation and virus destruction.

## 1. Introduction to Begomoviruses, Whiteflies and Circulative Transmission

Begomoviruses (genus *Begomovirus*, family *Geminiviridae*) are small ssDNA viruses vectored in a circulative persistent manner by the whitefly *Bemisia tabaci* (genus *Homoptera*, family *Aleyrodidae*). *B. tabaci* disseminates the viruses very efficiently, causing important damage to vegetables, staple food crops and ornamentals, worldwide [1]. The interactions between begomoviruses, plants and whiteflies have been the subject of many studies for the last fifty years. In this review, we follow the major events occurring during begomovirus circulation, from acquisition to transmission, and their interaction with organs and tissues of their insect vector. We have based our discussion mainly on the triangular relationship between the tomato yellow leaf curl virus (TYLCV) family, the vector *B. tabaci* whitefly species complex and the infected tomato (*Solanum lycopersicum*).

Begomoviruses have either two (bipartite, DNA-A and DNA-B) or one (monopartite, DNA-A-like), circular ssDNA genomic molecules of about 2800 nucleotides each. Each is separately encapsidated in a 22 nm × 38 nm geminate particle assembled from 110 copies of a single type of protein, the coat protein (CP). The virion strand of monopartite begomoviruses such as TYLCV encodes two genes, *V1* and *V2*; the complementary-virion strand (synthesized during the viral DNA replication) encodes four genes, *C1*–*C4* [2]. *V1* encodes the CP; the other genes encode proteins involved in virus replication and spread, as well as neutralizing plant defenses [3]. Bipartite begomoviruses possess a DNA-A (with functions similar to monopartite viruses) and a DNA-B, which contains two genes (one on the genomic strand, the other on the complementary genomic strand) with roles in virus intra- and inter-cellular movement.

TYLCV is one of the most-studied complexes of begomoviruses [1,4]. TYLCV is a monopartite begomovirus, except for TYLCV from Thailand, which has two genomic components [5]. The TYLCV complex comprises several species, such as TYLCV-Mild (TYLCV-Mld), tomato yellow leaf curl Sardinia virus (TYLCSV) and tomato yellow leaf curl China virus (TYLCCNV) [6]. TYLCV is one of the most economically-important plant viruses causing significant damage to tomato cultures worldwide [1]. Aggravated TYLCV disease symptoms in tomato are often associated with a TYLCV satellite DNAβ as described in China (TYLCCNV DNAβ) [7] and in Jordan (TYLCV DNAβ) [8].

The whitefly *B. tabaci* is a sibling species group comprising more than 30 species, or biotypes, including the widespread B (or Middle East-Asia Minor 1 (MEAM1)) and Q (or Mediterranean (MED)) [9,10]. *B. tabaci* may ingest viruses belonging to at least five genera: *Begomovirus*, *Carlavirus*, *Crinivirus*, *Ipomovirus*, *Torradovirus* [11]. The genetic material, the shape of the virion and the mode of transmission of these viruses by whitefly species are very different. Most studies have focused on the ingestion and transmission of begomoviruses (genus *Begomovirus*, family *Geminiviridae*) and criniviruses (genus *Crinivirus*, family *Closteroviridae*).

**Figure 1:**
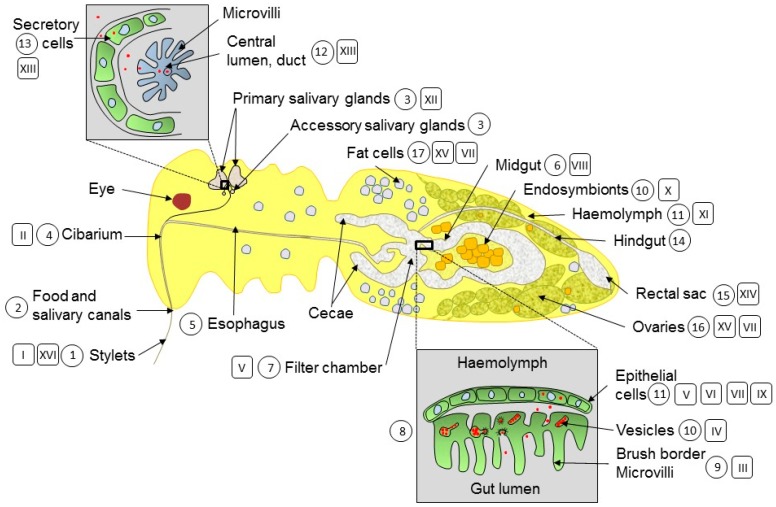
Schematic drawing of a female whitefly longitudinal cross-section. The main organs and cells involved in begomovirus translocation (Arabic numerals in a circle) and the main processes affecting the viruses (Roman numerals in a rectangle) are shown in the drawing. The important virus translocation sites in the midgut and the primary salivary glands are enlarged. Organ and cells: 1. stylets; 2. maxillary stylet containing the food and salivary canals; 3. primary and accessory salivary glands; 4. cibarium; 5. esophagus; 6. filter chamber; 7. midgut; 8. section across the filter chamber; 9. microvilli; 10. bacteriosome and endosymbiotic bacteria; 11. haemolymph; 12. salivary gland lumen; 13. salivary gland secretory cells; 14. hindgut; 15. rectal sac; 16. ovaries; 17. fat cells. Processes: I. virus ingestion; II. cibarium: discrimination non-circulative and circulative viruses; III. entry of virus via microvilli; IV. endocytosis; V. virus accumulation; VI. transcription, replication, virion formation; VII. autophagy; VIII. interactions virus CP-whitefly proteins; IX. virus aggregation; X. GroEL production; XI. interaction endosymbiotic GroEL-begomovirus CP; XII. release of virion from GroEL near primary salivary glands; XIII. secretion of begomovirus in primary salivary glands, from secretory cells into central lumen; XIV. virus excretion with honeydew; XV. virus invasion of fat cells and ovaries; XVI. virus egestion and transmission to plants.

The circulative pathway of begomoviruses in *B. tabaci* (Figure 1) and the velocity of translocation are known in its broad lines [12,13]. *B. tabaci* ingests TYLCV with its stylets while feeding on the phloem of infected plants (acquisition access period (AAP)). Virions pass along the food canal and reach the esophagus 10 min after the beginning of the AAP and the midgut after 40 min. They cross the filter chamber and the midgut area into the haemolymph after 90 min. From there, they translocate into the primary salivary glands (PSGs) where they can be detected 4–7 h after the onset of feeding. Then, virus is egested with the saliva along the salivary canal into the plant phloem (inoculation access period (IAP)). TYLCV transits with the same velocity in females and males [14]. It takes a minimum of 8 h (latent period) from the beginning of ingestion until TYLCV can be transmitted to tomato plants and produce disease symptoms 2–4 weeks thereafter [13]. Bipartite begomoviruses such as squash leaf curl virus (SLCV) follow the same route with a similar velocity [12]. TYLCVs are able to invade tissues other than those of the circulative path, such as the female reproductive system and fat bodies [15].

The amount of TYLCVs accumulating in individual 4–7 day-old adult *B. tabaci*, B or Q biotypes, during continuous feeding on infected tomato plants reaches a limit after 12–48 h, not exceeding about 600 million viral genomes. Amazingly, this amount remains nearly constant while the insects continue to feed on infected plants, and they have the potential to ingest about 600 million viruses every 24 h, implying mechanisms controlling the viral amounts in the insect [16,17,18,19]. Similar kinetics of virus accumulation during *B. tabaci* B feeding on infected plants were described for the bipartite SLCV [20] and watermelon chlorotic stunt virus (WmCSV) [21] and the monopartite papaya leaf curl China virus (PaLCuCNV) [22]. After *B. tabaci* B fed for a 48-h AAP on tomatoes infected with TYLCV, TYLCSV or TYLCCNV and they have been transferred to a virus non-host plant, the amounts of virus in the insects decreased by 1–2% per day [17,19,23]. This phenomenon was also observed with *B. tabaci* Q, Asia I and Asia II [22]. Although they retained substantial amounts of virus, the whiteflies gradually lost their ability to infect tomato plants, maybe because the aging insects are not as active as they once were [24,25,26]. One may also speculate that with time, the virus accumulates changes in their genome and structure detrimental to their infectivity.

Although the time-course pattern of virus ingestion and accumulation in whiteflies during feeding is a general feature, the velocity of ingestion and the amounts of ingested viruses may differ. Indeed, the biotype of the insect, age and gender and the virus concentration in the infected source plants have been shown to be factors that influence the kinetics of virus accumulation in the insect and the ability to transmit the virus to plants and induce disease.

Begomoviruses, while translocating in the *B. tabaci* circulative path, have to cross several barriers and checkpoints successfully, while interacting with would-be receptors and other whitefly proteins. In this review, we discuss the different sites begomoviruses need to cross to complete a successful circular infection. Figure 1 presents a schematic drawing of a whitefly longitudinal cross-section. The main organs involved in begomovirus translocation (Arabic numerals in a circle) and the main processes affecting the viruses (Roman numerals in a rectangle) are shown in the drawing. The virus translocation sites in the midgut and the primary salivary glands are enlarged.

## 2. The CP: A Key to Begomovirus Translocation in *B. tabaci*

Located at the surface of the virion, the CP is in contact with various insect tissues, from ingestion to egestion. Analysis of the CP of non-transmissible begomoviruses, of natural non-transmissible mutants of otherwise transmissible begomoviruses, and of in vitro mutated CP allowed identifying the amino acid stretches recognized by would-be receptors, ensuring virus translocation. Neutralizing the CP binding properties by feeding whiteflies through membranes with virion preparations together with CP antibodies was used to demonstrate that virions bind to insect putative receptors. The CP interacts at several sites with *B. tabaci* tissues to ensure further translocation until egestion.

The CP discriminates between begomoviruses that are able to cross the gut/haemolymph barrier and those that do not. Changing a few residues in the region of the CP between amino acids 129 and 152 restored infectivity of a natural non-transmissible TYLCSV isolate [27,28]. This region was homologous to that of abutilon mosaic virus (AbMV), a bipartite begomovirus that has lost its ability to be transmitted by *B. tabaci* to plants. AbMV is ingested, but is unable to cross the gut barrier into the haemolymph. Mutagenesis of the AbMV CP domain from amino acid 123–174 changed AbMV from non-transmissible to transmissible by *B. tabaci* [29].

The CP also discriminates between viruses that penetrate PSGs and are egested and those that are unable to do so, even if they are closely related [30]. For example, *B. tabaci* B efficiently transmits TYLCV and TYLCCNV, but *B. tabaci* Q is unable to transmit TYLCCNV. While TYLCV translocates in the Q PSGs until it reaches specific cells along the salivary duct and is egested, TYLCCNV remains in PSGs circumference cells until it disappears. After swapping a 483-bp fragment (positions 212–694) of the TYLCCNV *CP* gene with a 486-bp fragment (positions 215–700) of the TYLCV *CP* gene, the chimeric TYLCCNV accumulated in the PSGs of Q whiteflies, while the chimeric TYLCV was nearly undetectable. Therefore, it appears that the CP region mediating PSG recognition was also that necessary for crossing the gut wall into the haemolymph [27,31].

The CP is also essential for virus invasion of the female reproductive system of *B. tabaci* B and Q. TYLCV and TYLCSV can be transovarially transmitted to progeny, with variable levels of infectivity [32,33,34]. Entry of TYLCV into the developing eggs was mediated by the interaction of CP with the egg vitellogenin. In contrast, PaLCuCNV CP did not interact with whitefly vitellogenin, and this virus was not transovarially transmitted by whiteflies. However, when the PaLCuCNV CP amino-acid stretch 81–221 was replaced with the TYLCV amino-acids stretch 81–222 (33 amino acids changes amongst the two), and vice versa, the recombinant PaLCuCNV became transovarially transmissible and the recombinant TYLCV non-transmissible. Both wild type and CP-recombinant viruses were readily immune-detected in the midgut and in the salivary glands and were transmitted to plants [34].

These results indicated that the very CP region necessary for crossing the gut into the haemolymph was also necessary to penetrate the PSGs and the ovaries. This implies that the potential receptors of TYLCV in the gut, salivary glands and eggs may be identical or related.

## 3. Organs, Tissues and Cells Visited by Begomoviruses Circulating in *B. tabaci*

### 3.1. Penetration of the Stylet into the Phloem of Infected Plants

#### 3.1.1. The Plant Virus Source

Most begomoviruses are phloem-limited and are restricted to the vascular system; a few, like the bipartite *Bean dwarf mosaic virus*, also invade mesophyll tissue [35]. *B. tabaci* ingests begomoviruses by inserting its stylets in the vascular system of infected leaves. Virus amounts in an infected plant vary with the age of the tissues; therefore, the amounts of virus ingested and the kinetics of virus accumulation may depend on the virus concentration in the target leaves. In TYLCV-infected tomato, 15 days after whitefly-mediated inoculation at the six-leaf stage, the highest concentrations of viral DNA were found in the shoot apex (9 ng/μg plant DNA, equivalent to 5.4 × 10^6^ viral genomes/ng plant DNA) and in the three younger leaves (about 4 ng/μg DNA). Much less viral DNA was present in the older leaves (0.01 ng/μg DNA) and very little in the cotyledons (about 0.003 ng/μg plant DNA) [36]. Furthermore, individual *B. tabaci* B feeding on the same leaf for the same time period could ingest different amounts of virus [17].

In another instance, it was shown that the amount of virus uptaken by *B. tabaci* B was dependent on the virus concentration in the plant source and not on the virus itself [21]. The concentration of the bipartite *Watermelon chlorotic stunt virus* (WmCSV) in watermelon was about 10-times higher than that of TYLCV in tomato (70 × 10^5^ vs. 6 × 10^5^ genomes per ng plant DNA); as a result, after five days of AAP, whiteflies contained about 10-fold more WmCSV than TYLCV (about 50 × 10^6^ vs. 4 × 10^6^ viral genomes per insect). Membrane feeding with media containing the same concentrations of WmCSV and TYLCV (300 μg of purified virus per mL) showed that after two days, whiteflies contained approximately 1 × 10^6^ virus genomes, whether WmCSV or TYLCV.

It is important to note that virus concentrations in plants are usually measured in leaf homogenates, not in the phloem, and in groups of insects, not individuals. Nonetheless, these results show that the amount of virus ingested by *B. tabaci* is more dependent on the virus concentration in the plant than on the virus identity.

#### 3.1.2. Virus Ingurgitation

*B. tabaci* feeds on phloem sap by inserting its stylets into the vascular tissue (Figure 1,1,I) [37]. The stylet bundle is composed of three joined stylets: the maxillary stylet, which contains the interlocked food canal (through which phloem sap is ingested) and the salivary canal (through which saliva is injected into the plant), as well as two mandibular stylets (Figure 1,2) [38]. Before plant penetration with their stylets and during stylet movement through the apoplast, whiteflies secrete gelling saliva, forming a saliva sheath around the stylet. After penetrating a sieve tube, whiteflies secrete watery saliva prior to ingestion. Both saliva types are produced in the salivary glands (Figure 1,3) [39]. Ingestion is facilitated by hydrostatic pressure in the thieve tubes and stylet [40]. Ingestion of TYLCV is quite rapid. The virus is detectable by PCR in the head of whiteflies 5 min after the beginning of the AAP [41]. This correlates with the time it takes until stylets reach the sieve elements and start phloem ingestion, as determined by the electrical penetration graph (EPG) technology [42].

### 3.2. The Cibarium (Foregut) Discriminates between Non-Circulative and Circulative Viruses

The food canal empties into the precibarium and cibarium (Figure 1,4,II). They are the primary taste organs of the whitefly [43]. The cibarium joins the esophagus, which runs along the dorsal side of the thorax before entering the filter chamber [44]. The cibarium discriminates between circulative viruses, which will continue to move along the digestive tract, and non-circulative viruses, which will stop translocating and will accumulate in this organ.

In addition to begomoviruses, *B. tabaci* is able to ingest viruses belonging to at least four genera: *Carlavirus*, *Crinivirus*, *Ipomovirus* and *Torradovirus*, which are transmitted in a non-circulative non-persistent manner [11]. Most studies have focused on the ingestion and transmission of begomoviruses and criniviruses (genus *Crinivirus*, family Closteroviridae). Begomoviruses and criniviruses are transmitted by the same *B. tabaci* species, A, B and Q, albeit with different efficacies [45]. In addition, criniviruses can be transmitted by other whitefly species such as *Trialeurodes vaporariorum* and *T. abutiloneus* [46], whereas begomoviruses can be ingested, but not transmitted by T. vaporariorum.

Begomoviruses are transmitted exclusively by *B. tabaci* in a circulative, persistent manner. Once the virus is ingested, it translocates through the digestive system, the haemolymph, the salivary glands and is finally egested in the phloem. Following a 24-h AAP, the insect is able to transmit the virus for the rest of its life. In comparison, once ingested, criniviruses are retained for several days only in the insect cibarium during which they can be inoculated when the vector feeds on the phloem of a plant before losing transmission abilities [47].

The genetic material and the shape of the virion of these viruses are very different. Begomoviruses have either one or two ~2800 nucleotide circular ssDNA genomes, each encapsidated in a geminate particle (see Figure 1). Criniviruses such as the *Lettuce infectious yellows virus* (LIYV) and *Tomato chlorosis virus* (ToCV) appear as flexible filamentous rods with a diameter of 10–13 nm and a length of 700–900 nm. They have a genome split into two linear ssRNA molecules of about 15,000–20,500 nucleotides in total [48,49]. RNA 1 encodes proteins involved in viral replication; RNA 2 encodes a number of proteins including a minor (CPm) and a major (CP) coat protein. The CP encapsidates most of the virion particle, while the CPm encapsidates the RNAs 5’ end. The two RNAs are separately encapsidated.

CPm plays a critical in binding. Feeding whiteflies with a LIYV CPm antibody (but not a CP antibody) neutralized LIYV transmission [50]. It is likely that the antibody prevented CPm from binding to putative receptors in the insect cibarium. CPm mutagenesis could also impair the transmission of LIYV [51].

These different modes of transmission by the same insect imply the involvement of a still unknown selective mechanism controlling translocation of virus in the body of the vector. Transcriptome comparison of *B. tabaci* feeding on TYLCV- and on ToCV-infected tomato plants showed that clusters of unidentified genes were differentially expressed during the first three days of virus ingestion. They may represent genes that facilitate the specific mode of retention and transmission of ToCV and TYLCV and perhaps of other semi-persistent and circulative viruses [52].

### 3.3. Esophagus and Entry into the Guts

The esophagus joins the cibarium and runs along the dorsal side of the thorax before entering the filter chamber (Figure 1,5,7). The virus starts to be detected in the thorax of some insects after 10 min and in the abdomen after 25 min. In some females, part of or the entire midgut is pushed into the thorax by the developing ovaries, leaving only the hindgut in the abdomen [44,53]. The esophagus is lined with a cuticular intima making it an unlikely site of virus entry into the haemolymph.

### 3.4. Guts, Midgut and Filter Chamber

The midgut, especially the filter chamber (Figure 1,6,7), is the site of numerous events that affect the translocation and transmission of begomoviruses, their amount and likely their replication and expression. It is also the site where processes and proteins, which are beginning to be uncovered, affect the amount of virus associated with the insect and impact transmission to plants.

#### 3.4.1. Accumulation of Begomovirus in the Midgut and Filter Chamber

The filter chamber is formed by a sheath of thin epithelial cells. The internal surface of these cells faces the continuous lumen of the filter chamber. An inner layer of epithelial cells with extensive microvilli surrounds the lumen of the ileum, on the opposing side of the continuous lumen. The internal esophagus expands within the filter chamber where it is united with the continuous lumen, extends into the connecting chamber, cecae and descending and ascending midguts (Figure 1,8) [44]. The highly convoluted structure of the filter chamber with the presence of large Malpighian-like cells inside this structure suggests that it has a role in filtering food material [44].

Putative begomovirus receptors may be found in the midgut of *B. tabaci*. Indeed, midgut (within and outside the filter chamber) and internal ileum epithelia have a brush border at the apical membrane ideal for viral endocytosis (Figure 1,8) [14]. TYLCV CP was immuno-localized associated with microvilli of the gut lumen and with the gut epithelial cells (Figure 1,9,III), pointing to the virus translocation route through the gut wall and to a putative site of long-term virus storage [14,54]. Virions are transported through the cytoplasm of the filter chamber epithelial cells in vesicles that fuse with the basal plasma membrane, releasing particles between the membrane and the basal lamina (Figure 1,10–11,IV) [14,44]. Similar vesicles were described in the TYLCV non-vector whitefly *T. vaporariorum*; however, they were devoid of virus [55].

CP immunolocalization has shown that TYLCVs concentrate in the digestive tract, especially the midgut and the filter chamber (Figure 1,7,V) [56,57,58]. Other begomoviruses, such as the bipartite WmCSV, also concentrate in the midgut [57]. Examining the repartition of begomoviruses in sites along the circulative transmission by qPCR showed that once the amount of virus in an insect has reached optimal amounts after AAPs of 12–48 h, an overwhelming proportion is associated with the digestive tract and stays this way, likely for the remainder of the life of the insect.

In one set of investigations [21], the amounts of TYLCV were measured in organs dissected out of *B. tabaci* B. Three–five day-old whiteflies were caged for 5 days with infected tomatoes. Then, the insects were raised for two more days on virus non-host cotton plants. At the end of this treatment, individual midguts contained 6–50-times more than the haemolymph and 100-times more virus than the PSGs. In a parallel experiment performed with the same whitefly population feeding on WmCV-infected watermelons, individual midguts contained about 10–75-times more virus than the haemolymph and about 150-times more virus than the PSGs.

In another set of experiments [18], female *B. tabaci* B were reared for 20 days on tomato plants infected with either TYLCV or TYLCV-Mld. Then, the insects were divided into abdomen (with the guts), thorax (including the salivary glands), head (with salivary canal and stylet bundles) and haemolymph. The abdomen contained 200–800 million TYLCV genomes, the thorax and head about one-tenth this amount and the haemolymph about one hundredth the amount in the abdomen. The amount of TYLCV-Mld was about one-tenth the amounts of TYLCV in all four compartments studied. In a parallel experiment, the whiteflies were reared for 3 days on infected tomato, followed by 20 days on virus non-host cabbage plants. At this time, the amounts of TYLCV and TYLCV-Mld in the head, thorax and abdomen were similar to those present after 20 days of continuous virus ingestion. These results suggest long-term storage of TYLCV in the guts, as well as a stable distribution of the virus in the insect organs.

#### 3.4.2. Transcription and Replication

Replication was recently investigated in several instances. In the first case [59], *B. tabaci* B, 4–5 days after emergence, were first caged with TYLCV-infected tomato seedlings for 8 h AAPs, then transferred to cotton seedlings. The amounts of viral RNA and DNA in the insects were measured thereafter for up to 28 days. The amounts of *V1*, *V2* and *C3* transcripts increased 2–5-fold up to 1–3 days and decreased to basal levels thereafter. The amount of viral DNA in whole insects increased several fold during the first days on cotton and then decreased until they were barely detectable after 2 weeks. The amounts of TYLCV DNA in the head (with the salivary glands), the thorax (with the esophagus) and the abdomen (with the guts) increased up to three times, peaking after 2 days and decreasing thereafter, reaching initial amounts after 7 days. In dissected midguts, viral DNA amounts increased about seven-fold, peaking at 3–4 days, remaining stable for another 1–2 days and decreasing to initial levels after 6 days. FISH confirmed the accumulation of viral DNA and viral transcripts in the whitefly midguts. The complementary viral genome strand was detected in the nuclei of midgut epithelial cells, supporting the formation of a viral dsDNA replicative form and suggesting that midgut cell nuclei are the sites of virus replication (Figure 1,11,V,VI). Moreover, the immunodetection of CP implied the formation of newly-assembled virions. Interestingly, the application of sub-lethal doses of the pesticides acetamiprid and imidacloprid prevented the inhibition of replication. Following 8 h AAP on TYLCV-infected tomato, the insects were caged for 5 days with cotton leaves treated with the pesticides. During this period, the virus levels increased steadily up to five-fold, without the decrease observed after 2 days in non-treated controls. This pattern was not observed after the viruliferous whiteflies were raised for five days at 35 °C on cotton seedlings. FISH and immunodetection analyses confirmed the presence of the molecules associated with TYLCV replication under insecticide stress.

In the second case [60], whiteflies were given a 6-h AAP on TYLCV-infected tomato plants; then, they were reared on cotton plants for the next 12 days. Nearly undetectable for the first 4 h on cotton, a substantial amount of CP accumulated after 12 h and peaked at 48 h then slowly decreased to initial levels at 12 days. The amounts of viral genomic DNA paralleled those of the CP, increasing steadily, peaking at 48–96 h to about 20-fold the initial levels, then gradually decreasing until it was barely detectable at 12 days. The expression of genes on the genomic strand (*CP* and *V2*) and on the complementary strand (*C3*) also increased several fold and then decreased. Transcript accumulation and decay were correlated with the TYLCV-induced upregulation of the autophagy pathway (see below). Similar results were obtained with TYLCCNV, a monopartite begomovirus with a satellite DNAβ [7]. However, unlike TYLCV and TYLCCNV, the monopartite begomovirus *Papaya leaf curl China virus* (PalCuCNV) was unable to replicate and activate the whitefly autophagy pathway. The genomic DNA of PalCuCNV rapidly decreased and remained at a very low level thereafter, suggesting that this virus cannot replicate in the whitefly.

In the third experiment, the accumulation of TYLCV-Mld from Japan in the vector *B. tabaci* B and the non-vector *T. vaporariorum* was measured during 14 days of AAP of on infected tomato plants [61]. The kinetics of virus accumulation was similar in the two whitefly species. After 1 day, at the time insects accumulated about the same amount of virus, the insects were transferred to virus non-host cucumber plants. The amount of virus in *T. vaporariorum* remained constant for the 14 days of the experiment, while in *B. tabaci*, it increased about five times during the next 6 days, then decreased to levels present at the beginning of feeding on cucumber. Hence, TYLCV-Mld may replicate in *B. tabaci*, but not in *T. vaporariorum*.

On the other hand, two studies concluded that there was no evidence for TYLCV and TYLCV-Mild (TYLCV-Mld) replication in the whitefly vector. In the first experiment [18], the kinetics of accumulation of the two viruses in *B. tabaci* B constantly feeding on infected tomato plants were almost identical. A rapid increase was observed during a 24-h AAP, and each insect contained about 100 million viral genomes. These amounts increased slightly, if at all, with time thereafter. In another experiment, the insects were transferred to a non-host plant after AAPs of 6 and 48 h. No significant changes in the virus amount per insect were detected. The complementary DNA strand of TYLCV and TYLCV-Mld was present in insects from the first day of AAP, accumulated progressively over the 20 days of AAP, then decreased. It was hypothesized that this complementary strand was ingested from the infected plant and allowed a minor and transient replication in insects, before being degraded. The authors suggested that the stability of the insect viral content could be the result of preservation mechanisms without replication, rather than an equilibrium between virus replication and degradation.

In the second experiment [62], to prevent the acquisition of viral ssDNA and the dsDNA replicative form from plants that may stick to the capsid, *B. tabaci* B and Q were membrane-fed with DNAase I-treated purified TYLCV and TYLCV-Mld virions. Despite these precautions, both ssDNA and dsDNA were found associated with the particles. Moreover, after virion ingestion, whiteflies were maintained on an artificial diet to eliminate possible plant replication factors. Despite these precautions, viral ssDNA and dsDNA were detected in whiteflies. The ratio of virion/complementary strand remained approximately constant for 4 days of feeding on the artificial diet, indicating that replication was not taking place.

#### 3.4.3. Autophagy

Autophagy is a part of the cellular immune responses to virus infection in mammals [63]. In plants, autophagy is also induced by begomoviruses such as TYLCV [64] and TYLCSV [65]. Feeding of *B. tabaci* B on TYLCV-infected tomato plants activated the insect autophagy pathway [60]. After 6 h AAP on TYLCV-infected tomato, autophagy activation was studied after the transfer of the viruliferous whiteflies on the virus non-host cotton plants (time 0 h). At 0 h, the insects have ingested approximately 30 million viral genomes equivalent, one-twentieth of the 600 million genome threshold accumulated during continuous feeding. During the first 4 days following transfer on cotton, the amount of TYLCV DNA in individual whiteflies steadily increased, reaching 25-fold the initial amount and decreasing thereafter to basal levels after 12 days. Autophagy paralleled this decrease. The time course of autophagy induction and decay coincided with the onset and cessation of TYLCV DNA replication and gene transcription (see Figure 1). Western blot and immune-fluorescent localization in the insect digestive tract showed that the autophagy marker protein ATG8-II, which was not detected for the first 24 h, increased at 48 h and then gradually decreased until reaching initial levels (at 0 h) at 12 days. The expression of the autophagy-related genes *Atg3*, *Atg9* and *Atg12* also increased after 24–48 h on cotton, then decreased to the initial levels (at 0 h). The number of autophagosomes in the midgut of the viruliferous whiteflies increased and decreased with the same time-course. Inhibiting autophagy with 3-methyladenine induced an increase in TYLCV gene transcription and in virus transmission. Similar results were obtained with TYLCCNV. Immunofluorescence staining of autophagic signals and electron microscopy of autophagosome formation suggested that autophagy may play critical roles in the whitefly midgut, a site where TYLCVs are most abundant (Figure 1,11,VII).

Taken together, these experiments indicate that increasing amounts of TYLCV due to virus replication in whiteflies activate autophagy, which in turn suppresses replication and destroys the virions. TYLCV proteins may be degraded by whitefly proteases, ubiquitin 26S proteasome and autophagy [66,67]. Viruses such as PalCuCNV that do not replicate in whiteflies do not activate the autophagy pathway [60]. The replication-autophagy correlation was strengthened by examining the begomovirus non-transmitter *T. vaporariorum*. In this insect, TYLCV is ingested but does not penetrate the gut barrier and stays in the gut lumen. After a 6-h AAP on TYLCV-infected tomato and transfer to cotton plants, the amount of viral DNA did not increase, and autophagy was not detected.

Autophagy was not appraised in whiteflies continuously feeding on TYLCV-infected plants. Nonetheless, it is possible that autophagy is a major factor in achieving the threshold and maintaining the amount of virus approximately constant, despite continuous virus ingestion.

#### 3.4.4. Whitefly Proteins Interacting with Begomovirus Proteins in the Guts

Several proteins have been shown recently to bind to TYLCV in the midgut and thereby to influence TYLCV transmission (Figure 1,6,VIII). *Hsp70* was upregulated in viruliferous whiteflies. HSP70-TYLCV interaction correlated with a decrease in TYLCV transmission [57]. *Hsp70* transcripts increased upon ingestion and retention of TYLCV and SLCV. TYLCV CP and HSP70 interacted in vitro and co-localized within midgut epithelial cells [57]. Oral ingestion of anti-HSP70 antibodies together with TYLCV virions showed an increase in TYLCV transmission. It was suggested that HSP70-TYLCV interaction mediates the degradation of virions.

A midgut protein (coined MGP) was identified using the CP of the begomoviruses *Tomato leaf curl New Delhi virus* (ToLCV) and *Cotton leaf curl Rajasthan virus* (CLCuV) as bait in a yeast two hybrid screen [68]. MGP was localized mainly in the gut epithelial layer. Oral ingestion of anti-MGP antibodies resulted in a 2/3 decrease in transmission of ToLCV or CLCuV, thus indicating the importance of MGP in the virus-vector interaction process [68].

From the three *B. tabaci* peptidyl-prolyl isomerases protein genes (PPIases or cyclophilins Cyps) *CypB*, *CypD* and *CypG*, only the expression of *CypB* was upregulated by two-fold in viruliferous *B. tabaci* B. CypB and TYLCV CP formed complexes that could be resolved in vitro [58]. In addition, CypB and TYLCV CP co-localized in the filter chamber, cecae and midgut indicated that these interactions may mediate virus translocation in the haemolymph. Moreover, CypB and TYLCV CP co-localized in the PSGs and in oocytes. Oral feeding of whiteflies with anti-CypB antibodies induced a decrease in TYLCV transmission of about 43% [58]. Altogether, CypB may help TYLCV translocate from the gut into the PSGs. CypB may also play a role in the invasion of the female reproductive system by TYLCV and subsequently in the transovarial transmission of the virus [32,33,34].

A peptidoglycan recognition protein gene (PGRP) from *B. tabaci* B (coined *BtPRPG*) was upregulated upon ingestion and accumulation of TYLCV and TYLCCNV, but not of the monopartite *Tobacco curly shoot virus* TbCSV [69]. *BtPGRP* was expressed in several tissues at all life stages from egg to adult. PGPR and TYLCV CP interactions were detected in vitro by immunocapture PCR and in situ by fluorescent immuno-detection in the midguts of viruliferous whiteflies. Taken together, the *BtPGRP* gene is involved in whitefly immunity, as well as in begomovirus transmission [69].

#### 3.4.5. Storage in the Digestive Tract

The vast majority of the virus ingested during feeding remains in the digestive tract, especially in the filter chamber and midgut (Figure 1,6,7,V). Only a very small amount of the virus crosses to the haemolymph, reaches the salivary glands and is egested into plants (see Figure 1). Therefore, two questions arise: Why is it that not all of the viral particles cross into the haemolymph, and where and how are they stored to avoid degradation? In the insect body, viral particles and viral proteins may be considered as intruders by the host protection machinery. The capacity of *B. tabaci* to degrade the six TYLCV proteins is very high. Ultracentrifuge fractionation of whitefly proteins indicated that the highest proteolytic activities (26S proteasome and autophagy) were associated with fractions containing soluble proteins and much lesser in fractions containing midsize and large protein aggregates [66]. It was hypothesized that TYLCV could confront host degradation by sheltering in small/midsized aggregates, where viral proteins are less exposed to proteolysis (Figure 1,11,IX). Although not yet established, aggregates may serve as virus factories reminiscent of those in infected tomato plants [67]. A similar phenomenon was described in *Cauliflower mosaic virus* (CaMV)-infected *Arabidopsis* plants [70]. CaMV forms virus factory-like aggregates that protect the virus against autophagic destruction and promote virus multiplication, thereby potentially enhancing virus uptake by the aphid vector.

### 3.5. Gut-Haemolymph Barrier

To be transmitted, begomoviruses need to cross the gut haemolymph barrier. This checkpoint was demonstrated to be genetically determined by selecting good and poorly TYLCV transmitting lines from *B. tabaci* B populations [21]. Compared to good transmitter isolines, in the poor transmitter virus, translocation from the midgut into the haemolymph was considerably delayed and reduced, pointing to a block at the gut/haemolymph barrier, similar to that in the non-transmitter whitefly *T. vaporariorum*.

TYLCV is unable to cross the midgut barrier of *T. vaporariorum* to enter the haemolymph and from there the salivary glands [14]. Immuno-localization of TYLCV-Mld in the vector *B. tabaci* B and the non-vector *T. vaporariorum* showed that the virus was present at the brush borders and in the surrounding luminal surface of the midgut epithelial cells of both whitefly species. However, it was exclusively found in the cytoplasm of the midgut cells of *B. tabaci*, not in that of *T. vaporariorum* [61].

Endocytosis was the major process serving to transport TYLCV across the *B. tabaci* midgut barrier (Figure 1,10,IV). Indeed, endocytosis is the most common path exploited by virus to enter its host [71]. TYLCV accumulated in vesicle-like structures in the midgut epithelial cells of the vector whitefly *B. tabaci*, but not in those of the non-vector whitefly *T. vaporariorum* [55]. Hence, in order to cross the gut/haemolymph barrier, TYLCV must first enter the midgut cells, likely by endocytosis. This route was confirmed by disrupting components involved in clathrin-mediated assembly at the cell surface and disassembly at the endosome such as clathrin heavy and light chain (CHC and CHL), dynamin (DYN), *rab5* and *rab7* associated with early and late endosome, respectively. In these experiments [72], whiteflies were orally fed through membranes with the chemical inhibitors chlorpromazine (a specific inhibitor of clathrin assembly at cell surfaces and disassembly at the endosome), dynasore (a specific dynamin inhibitor) and with antibodies and gene silencing dsRNA against CHC and rab5. These treatments resulted in an increase in the quantity of virus in the whitefly midgut and a decrease in the haemolymph relative to that of the whitefly whole body. Immune-fluorescence detection of virus in whitefly orally fed with endocytosis inhibitor followed by virus ingestion showed that virus accumulated in the midgut. These experiments confirmed that clathrin-mediated endocytosis plays a major role in TYLCV transport across the midgut barrier of *B. tabaci*.

### 3.6. Endosymbionts

Whiteflies and endosymbiotic bacteria have interacted for millions of years [73]. Endosymbionts are housed in cells called bacteriosomes (Figure 1,10). The different *B. tabaci* biotypes (or species) harbor the obligatory primary whitefly endosymbiont *Portiera aleyrodidarum* and a variety of facultative secondary symbionts such as *Arsenophonus*, *Cardinium*, *Fritschea*, *Hamiltonella*, *Rickettsia* and *Wolbachia*. Different biotypes in different geographical regions harbor different sets of secondary symbionts [74]. Only *Rickettsia* localizes outside the bacteriocytes and appears in most of the body cavity and infects the digestive, reproductive and salivary systems, where it can reach high concentrations [75,76]. *Rickettsia* was shown to be directly involved in begomovirus transmission. Comparing two *B. tabaci* B populations, one infected with *Rickettsia*, the other *Rickettsia*-free, showed that high levels of the bacterium in the midgut pushed the virus out of the gut into the haemolymph. The presence of *Rickettsia* was correlated with increased TYLCV transmission efficiency suggesting that the invasion of the midgut with *Rickettsia* accelerated virus translocation out of the midgut [77]. *B. tabaci* endosymbionts produce GroEL homologue proteins, which are secreted into the haemolymph and ensure virus transmission (see below).

### 3.7. Haemolymph

The haemolymph constitutes an open blood system, bathing the whitefly internal organs (Figure 1,11). Once begomoviruses cross the gut barrier, they are prone to destruction by the proteases and nucleases conspicuous in the haemolymph [78]. To protect the virus in this hostile environment, the CP of TYLCV interacts with a 63-kDa GroEL chaperone produced by the whitefly endosymbiotic bacteria and released into the haemolymph (Figure 1,11,X); GoEL is not detected in the digestive tract. The physical interaction between TYLCV CP and GroEL (Figure 1,11,XI) was shown by yeast two-hybrid, immune-capture PCR, protein pull-down assays and by oral feeding with GroEL antibodies [79,80,81]. The release of virions protected by GroEL occurs adjacent to the PSGs (Figure 1,3,XII) [79]. The role of the whitefly GroEL in ensuring TYLCV circulative transmission is similar to that first demonstrated for the transmission of luteoviruses by their aphid vector [82].

The GroEL, which protects TYLCV from Israel from degradation in the haemolymph of *B. tabaci* B, is produced by *Hamiltonella* [79]. The poor TYLCV transmitter biotype Q does not contain *Hamiltonella*. GroELs produced by *Wolbachia* and *Arsenophonus* in both B and Q biotypes poorly interact with the TYLCV CP, indicating that they play a minor role, if at all, in TYLCV transmission [81]. It is interesting to note that *B. tabaci* Asia II isolates from Rajasthan, India, contain the primary endosymbiont *Portiera,* but only the secondary endosymbiont, *Arsenophonus*. Hence, GroEL from *Arsenophonus* ensures the transmission of cotton leaf curl virus (CLCuV). The potential interaction between GroEL and CLCuV CP was demonstrated by pull-down, immunoprecipitation and yeast two hybrid assays [83].

The haemolymph and the viruses it contains can be shuffled by the movement of the guts. It was shown that the midgut loop is able to move through the abdomen-thorax constriction, especially in gravid *B. tabaci* females [44,53]. As a result, the gut is closer to the PSGs, and the distance begomoviruses have to cross in the haemolymph is much reduced, as shown for SLCV [53].

### 3.8. Primary Salivary Glands

The primary salivary glands (PSGs), not the accessory salivary glands, are involved in begomovirus circulation [44,53]. The PSG pair are kidney shaped and contain at least 13 cells of different sizes (Figure 1,3). Each primary gland has a central lumen that empties into a duct that joins the accessory salivary gland duct. Both ducts fuse to form the lateral salivary ducts that terminate as the salivary canal contained in the stylet and end at the stylet tip [14,44,53].

The cells around the secretory region of the PSGs (Figure 1,13,XII) play a role in the specificity of begomovirus transmission and have been shown to contain *B. tabaci* species-specific receptor-like elements that are able to discriminate between begomoviruses, even as similar as TYLCV and TYLCCNV [32]. While *B. tabaci* B efficiently transmits TYLCV and TYLCCNV, *B. tabaci* Q ingests and retains both viruses, but is unable to transmit TYLCCNV. The selective transmission of TYLCV/TYLCCNV by Q whiteflies was investigated by following the time course of virus penetration in the PSGs, using CP immunofluorescence of dissected glands. The viruses followed a specific path in the glands. Following a 4–6-h AAP, TYLCV CP spread to the entire PSG; at 12–24 h, virions gradually accumulated to the central region of PSGs; at 36–72 h, the viral CP was observed only in the few cells along the salivary duct (Figure 1,13,XIII). In comparison, at 4–12 h AAPs, TYLCCNV was distributed in cells in the boundary of Q PSGs and disappeared thereafter. Therefore, secretory cells in the central region of whitefly PSGs govern the transmission specificity of begomoviruses. It also suggests that possible receptors may reside in these cells and account for virus recognition and secretion through PSGs. The PSGs of Q whiteflies might lack some component in these specific cells that interact with TYLCCNV, resulting in no transmission. These results showed that TYLCCNV penetrated the B PSGs, but not those of Q; as a result, TYLCCNV was present in saliva from B, but not of Q. Hence, the accumulation of virions in the cells around the PSGs secretory region, control the specificity of begomovirus transmission [30].

The transcriptome of the salivary glands of *B. tabaci* Q was analyzed. Many transcripts represented genes involved in metabolism, protein processing and secretion and a number of putative secretory proteins that may play a role in whitefly feeding and salivation [84]. However, proteins playing a role (or blocking) in begomovirus transmission were not identified.

### 3.9. Hindgut and Rectal Sac

The rectal sac had a heavy intima lining. When filled, it could expand to a relatively large size compared to other portions of the hindgut (Figure 1,14,15). The epithelial cells were quite thin when the structure was expanded and had nuclei that were small relative to the midgut and filter chamber. It had a valve-like structure with large external muscles that presumably contracted to open the valve releasing honeydew via the rectum and the anus (Figure 1,15,XIV). SLCV was detectable by PCR in the honeydew 8 h after the beginning of the AAP [12].

### 3.10. Other Tissues: Fat Cells, Reproductive System

TYLCVs are present not only in the virus circulative pathway (stylet, guts, haemolymph and salivary glands), but also in other tissues such as the female reproductive system (Figure 1,16) and fat bodies (Figure 1,17) [15]. It is possible that TYLCV is restricted to these cells and does not invade other tissues, such as bacteriocytes, muscle and ganglions. Either these tissues were not thoroughly examined or they are protected from virus invasion by a still unknown mechanism. Virus invasion of cells from male whiteflies has not been documented.

Autophagy was shown to restrain TYLCCNV invasion of fat cells and ovaries (Figure 1,16,17,VII) [15]. In B and Q whiteflies, after an initial 24-h AAP on TYLCNV-infected tobacco and transfer to virus non-host cotton, the autophagy protein marker Atg8-II was detected after 5 days. These results indicated that autophagy was activated only after prolonged TYLCCNV associated with the insect. At 120 h, dissected ovarioles and fat body tissues were the sites of induced autophagic degradation activities. Concomitantly to the activation of autophagy, the number of whiteflies with detectable viral DNA in the ovaries and in the fat bodies decreased by about half, indicating that TYLCCNV invasion of the ovary and fat body tissues inactivated autophagy, which probably leads to the gradual decrease of the number of virions in these tissues.

Virus penetration of ovaries may explain TYLCV and TYLCCNV transmission to egg and to progeny [32,33,34]. To penetrate the developing egg, TYLCV CP needs to interact with the egg vitellogenin. In contrast, PaLCuCNV CP did not interact with whitefly vitellogenin, and this virus was not transovarially transmitted by whiteflies [34]. TYLCV accumulation was associated with a decrease in the whitefly fertility, as measured by the mean number of eggs laid per a female during [24] or after feeding on infected tomato and transfer to cotton [59]. The way TYLCV penetrates fat cells is unknown.

### 3.11. Egestion into the Plant Vascular System

Egestion is the reverse image of ingestion. Virus is egested into the vascular system of the plant together with the saliva (Figure 1,1,XVI). Transmission of TYLCV by *B. tabaci* B is very rapid. The virus is detectable in the leaf on which whiteflies are feeding within 5–30 min after the beginning of the inoculation access period [41].

After 12–48 h AAP on infected tomato plants (when whiteflies accumulated an optimum amount of virus) and were transferred to virus non-host plants, the amount of virus they carried decreases by 1–2% every day [17,23,24,25]. This decrease may be due to egestion into the phloem of the non-host plant during feeding. Postulating that this decrease is due solely to egestion and that it reflects the amount of virus inoculating tomato plants, one can estimate that during a 24 h IAP, whiteflies egest about 6–12 million viral particles, compared to the 600 million ingested.

## 4. Conclusions

Translocation of begomoviruses depends on their interaction with many *B. tabaci* tissues. At every stage along the circulative pathway, begomoviruses have to pass checkpoints located in the cibarium, the lumen/gut interface, the gut/haemolymph interface and the PSGs. At each location, the virus CP plays a major role in recognizing would-be receptors [27,28,29,30,31,34], allowing further virus translocation. Interestingly, it is the same amino acid stretch in the CP that is involved in the translocation of begomoviruses into the haemolymph and the PSGs, as well as in the invasion of the maturing eggs in the reproductive system, suggesting that these receptors are structurally related. Ingestion of CP antibodies has shown that it is possible to disturb the CP-receptor complex, thereby inhibiting virus translocation and diminishing virus transmission to plants. Delivery of small peptides designed to mimic the essential region of the CP (either expressed in plants or delivered by spraying) may block receptors and inhibit begomovirus translocation.

The identification of begomovirus receptors in *B. tabaci* has remained elusive, though the technical panoply of researchers has greatly extended in the recent years. Comparative transcriptome analyses and the availability of sequence of the genomes of *B. tabaci* B [85] and Q [86] biotypes have raised many expectations. Transcriptome analyses of viruliferous vs. non-viruliferous whiteflies have shown that TYLCVs infection modifies the expression of many genes [15,87,88]. The genes may not be those that play a direct role in virus circulative transmission and its regulation and control. Rather, they may be part of a general stress response induced by the insect facing virus invasion. The comparison of transcriptomes of *B. tabaci* organs such as guts [89] and salivary glands [84] may help identify common patterns of tissue-specific expression upon virus invasion. The transcriptome analysis of developing insect [87,90] may be more promising. Indeed, it is unlikely that the genes encoding the begomovirus receptors are transcribed during the very first minutes of virus ingestion and that the newly synthesized receptor proteins find their site of action almost immediately. Hence, receptors are likely present before virus ingestion; would-be virus receptors may exert additional, unrelated functions and are used opportunistically by the virus. Would-be receptors are likely to be formed during early development, from egg to early pupae. Unfortunately, the embryology of *B. tabaci* is not well known and has to be worked out.

The search for putative begomovirus receptors and for proteins regulating the translocation of begomoviruses in *B. tabaci* has witnessed new developments. Researchers have been able to select for poor begomovirus transmitters within whitefly populations [21]. In these insects, WmCSV and TYLCV were unable to cross the gut barrier into the haemolymph, a sine qua non condition for virus transmission. Since this property seems to be genetically controlled, it might be possible to multiply such whitefly populations to industrial levels in order to compete with begomovirus transmitters. Of course, this population might be diluted by mating with transmitter whiteflies, and heterozygous females may be good transmitters. This aspect ought to be experimentally determined and its potential as pest control assessed.

Recently, several whitefly proteins have been identified, which either increase or decrease begomovirus transmission. Most of these proteins have been immuno-located in the midgut, and some of them bind the CP. The events begomoviruses are subjected to, once ingested, emphasize the tissues and processes that are mobilized to control the virus amounts to avoid a virus overflow that might be lethal to the insect. Even, the invasion of organs other than those in the circulative pathway such as fat cells and ovaries are controlled and restrained. Recently, a gene encoding a knottin protein (*knot-1*) was identified as a possible regulator of virus amounts in *B. tabaci* [91]. Silencing *knot-1* increased by several orders of magnitude the quantity of virus ingested by the insects and transmitted to tomato plants. Hence, it seems that *knottin-1* is involved in restraining the amount of TYLCV particles associated with *B. tabaci* during the circulation of the virus in the insect body.

The recently published sequence of the genomes of the *B. tabaci* B [85] and Q [86] biotypes may help find additional genes that ensure a suitable live and let live equilibrium between begomoviruses and their whitefly host and may help to expand the knowledge of the interesting relationships between the *B. tabaci* vector and the begomovirus intruder. The sequence of the genome of the begomovirus non-vector *T. vaporariorum*, when available, will help focus our quest for genes related to vectoriality.
